# Supplementary material for: Dimethyl fumarate in patients admitted to hospital with COVID-19 (RECOVERY): a randomised, controlled, open-label, platform trial
Source: Nat Commun. 2024 Jan 31;15:924. doi: 10.1038/s41467-023-43644-x (PMC10831058; doi:10.1038/s41467-023-43644-x)
Supplement: Supplementary file 3 — Reporting Summary [file 41467_2023_43644_MOESM3_ESM.pdf]

nature portfolio

Corresponding author(s): Leon Peto

Last updated by author(s): Oct 13, 2023

Reporting Summary

Nature Portfolio wishes to improve the reproducibility of the work that we publish. This form provides structure for consistency and transparency in reporting. For further information on Nature Portfolio policies, see our Editorial Policies and the Editorial Policy Checklist.

Statistics

For all statistical analyses, confirm that the following items are present in the figure legend, table legend, main text, or Methods section.

n/a	Confirmed	
☐	☒	The exact sample size (*n*) for each experimental group/condition, given as a discrete number and unit of measurement
☐	☒	A statement on whether measurements were taken from distinct samples or whether the same sample was measured repeatedly
☐	☒	The statistical test(s) used AND whether they are one- or two-sided<br>*Only common tests should be described solely by name; describe more complex techniques in the Methods section.*
☐	☒	A description of all covariates tested
☐	☒	A description of any assumptions or corrections, such as tests of normality and adjustment for multiple comparisons
☐	☒	A full description of the statistical parameters including central tendency (e.g. means) or other basic estimates (e.g. regression coefficient) AND variation (e.g. standard deviation) or associated estimates of uncertainty (e.g. confidence intervals)
☐	☒	For null hypothesis testing, the test statistic (e.g. *F*, *t*, *r*) with confidence intervals, effect sizes, degrees of freedom and *P* value noted<br>*Give P values as exact values whenever suitable.*
☒	☐	For Bayesian analysis, information on the choice of priors and Markov chain Monte Carlo settings
☒	☐	For hierarchical and complex designs, identification of the appropriate level for tests and full reporting of outcomes
☒	☐	Estimates of effect sizes (e.g. Cohen's *d*, Pearson's *r*), indicating how they were calculated

Our web collection on statistics for biologists contains articles on many of the points above.

Software and code

Policy information about availability of computer code

Data collection	Baseline information was collected using a validated online randomisation system developed by trial progammers, and follow-up information used a commercial electronic data capture system developed for clinical trials (OpenClinica). Full details of the forms used and methods of data derivation are in the supplementary appendix.
Data analysis	Analyses were performed using SAS version 9.4 and R version 3.4.

For manuscripts utilizing custom algorithms or software that are central to the research but not yet described in published literature, software must be made available to editors and reviewers. We strongly encourage code deposition in a community repository (e.g. GitHub). See the Nature Portfolio guidelines for submitting code & software for further information.

Data

Policy information about availability of data

All manuscripts must include a data availability statement. This statement should provide the following information, where applicable:

- Accession codes, unique identifiers, or web links for publicly available datasets
- A description of any restrictions on data availability
- For clinical datasets or third party data, please ensure that the statement adheres to our policy

The protocol, consent form, statistical analysis plan, definition & derivation of clinical characteristics & outcomes, training materials, regulatory documents, and other relevant study materials are available online at www.recoverytrial.net. As described in the protocol, the trial Steering Committee will facilitate the use of the study data and approval will not be unreasonably withheld. Deidentified participant data will be made available to bona fide researchers registered with an

appropriate institution within 3 months of publication. However, the Steering Committee will need to be satisfied that any proposed publication is of high quality, honours the commitments made to the study participants in the consent documentation and ethical approvals, and is compliant with relevant legal and regulatory requirements (e.g. relating to data protection and privacy). The Steering Committee will have the right to review and comment on any draft manuscripts prior to publication. Data will be made available in line with the policy and procedures described at: https://www.ndph.ox.ac.uk/data-access. Those wishing to request access should apply for access via the Infectious Diseases Data Observatory (https://www.iddo.org/covid19/data-sharing/accessing-data), or complete the form at https://www.ndph.ox.ac.uk/files/about/data_access_enquiry_form_13_6_2019.docx
and e-mail to: data.access@ndph.ox.ac.uk

Research involving human participants, their data, or biological material

Policy information about studies with human participants or human data. See also policy information about sex, gender (identity/presentation), and sexual orientation and race, ethnicity and racism.

Reporting on sex and gender	For all participants in the RECOVERY platform trial, participant sex as documented in the medical records was recorded, which reflects usual practice in medial record keeping. The characteristics of the trial population are provided in table 1 and webtable 1. Subgroup analyses were not performed by sex in the DMF comparison, as the limited sample size meant there was insufficient statistical power to reliably detect an interaction in treatment effect by sex, and an appreciable risk of drawing false conclusions.
Reporting on race, ethnicity, or other socially relevant groupings	For all participants in the RECOVERY platform trial, participant ethnicity was recorded from NHS records. The characteristics of the trial population are provided in table 1 and webtable 1. Subgroup analyses were not performed by race or ethnicity in the DMF comparison, as the limited sample size meant there was insufficient statistical power to reliably detect an interaction in treatment effect by ethnicity, and an appreciable risk of drawing false conclusions.
Population characteristics	These are described in Table 1 of the manuscript
Recruitment	Patients admitted to hospital were eligible for the study if they had clinically suspected or laboratory confirmed COVID-19 and no medical history that might, in the opinion of the attending clinician, put the patient at significant risk if they were to participate in the trial. Eligible patients were approached by trial staff who explained the trial, and if they wanted to join they provided informed consent. Patients did not volunteer for the study, and were approached whilst in hospital. There are likely to be some differences between patients accepting and not accepting participation, but this would not bias the results of the randomised treatment comparison that is reported in this manuscript. Participants were not compensated for taking part in the trial.
Ethics oversight	The trial is conducted in accordance with the principles of the International Conference on Harmonisation–Good Clinical Practice guidelines and approved by the UK Medicines and Healthcare products Regulatory Agency (MHRA) and the Cambridge East Research Ethics Committee (ref: 20/EE/0101). The protocol and statistical analysis plan are included in the appendix (pp 63-139) with additional information available on the study website www.recoverytrial.net.

Note that full information on the approval of the study protocol must also be provided in the manuscript.

Field-specific reporting

Please select the one below that is the best fit for your research. If you are not sure, read the appropriate sections before making your selection.

[x] Life sciences [ ] Behavioural & social sciences [ ] Ecological, evolutionary & environmental sciences

For a reference copy of the document with all sections, see nature.com/documents/nr-reporting-summary-flat.pdf

Life sciences study design

All studies must disclose on these points even when the disclosure is negative.

Sample size	It was estimated that enrollment of at least 700 patients would provide 80% power (at 2p=0.05) to detect a common odds ratio of 0.67, even if 10% of participants discontinued study treatment before day 5 (based on day 5 outcome frequencies from the existing trial database). The final sample size was 713.
Data exclusions	All randomised participants were included in the interntion-to-treat analysis, in keeping with the pre-specified statistical analysis plan. Thee were no data exclusions.
Replication	No replication of the trial has been attempted by the investigators
Randomization	Patients were randomly allocated to groups as described in methods section 'randomisation and masking'
Blinding	Participants and local study staff were not masked to the allocated treatment. The trial steering committee, investigators, and all other individuals involved in the trial were masked to aggregated outcome data during the trial.

Reporting for specific materials, systems and methods

We require information from authors about some types of materials, experimental systems and methods used in many studies. Here, indicate whether each material, system or method listed is relevant to your study. If you are not sure if a list item applies to your research, read the appropriate section before selecting a response.

Materials & experimental systems

n/a	Involved in the study
☒	☐ Antibodies
☒	☐ Eukaryotic cell lines
☒	☐ Palaeontology and archaeology
☒	☐ Animals and other organisms
☐	☒ Clinical data
☒	☐ Dual use research of concern
☒	☐ Plants

Methods

n/a	Involved in the study
☒	☐ ChIP-seq
☒	☐ Flow cytometry
☒	☐ MRI-based neuroimaging

Clinical data

Policy information about clinical studies

All manuscripts should comply with the ICMJEguidelines for publication of clinical research and a completedCONSORT checklist must be included with all submissions.

Clinical trial registration	ISRCTN50189673; NCT04381936
Study protocol	It is present in the supplementary appendix, and on the trial website (link is provided in the paper)
Data collection	Between 2 March 2021 and 18 November 2021, 713 patients were enrolled into the trial at 27 UK hospital organisations. Participants were inpatients in acute medical wards, and data were collected onto online case report forms by clinical or research staff at the hospital.
Outcomes	The primary outcome was clinical status at day 5, as assessed on the ordinal scale. Secondary outcomes were: time to sustained improvement by at least one category on the ordinal scale from baseline (persisting for >1 day), time to discharge from hospital, S/F94 ratio at day 5, blood C-reactive protein at day 5, and improvement in clinical status by at least one category at day 10. The initial protocol specified day 5 S/F94 as the primary outcome and day 5 clinical status as a secondary outcome, but these were switched in October 2021 when it was realised that discharges before day 5 would lead to significant amounts of missing data for the S/F94 outcome. This decision was made by the trial steering committee whilst blinded to the results of the DMF comparison.<br>Subsidiary clinical outcomes were: use of ventilation and, separately, use of renal dialysis or haemofiltration, among patients not on such treatment at randomisation, and thrombotic events. Pre-specified safety outcomes were: flushing, gastrointestinal symptoms, transaminitis (peak alanine transaminase (ALT) or aspartate transaminase (AST) >3x upper limit of normal), acute kidney injury (peak creatinine >1.5x value at randomisation), cause-specific mortality, bleeding events, major cardiac arrhythmias, and non-coronavirus infections. Information on suspected serious adverse reactions was collected in an expedited fashion to comply with regulatory requirements.
